# Effects of Dual Wavelength Photobiomodulation on Osseointegration in Grafted Areas

**DOI:** 10.1002/lsm.70126

**Published:** 2026-03-27

**Authors:** Lucas de Sousa Goulart Pereira, Julia Raulino Lima, Elcio Marcantonio, Priscilla Barbosa Ferreira Soares, Suzane Cristina Pigossi, Guilherme José Pimentel Lopes de Oliveira

**Affiliations:** ^1^ Department of Periodontology School of Dentistry Federal University of Uberlândia—UFU Uberlândia Minas Gerais Brazil; ^2^ Department of Diagnosis and Surgery Araraquara School of Dentistry, Universidade Estadual Paulista (FOAr‐UNESP) Araraquara São Paulo Brazil

**Keywords:** bone substitutes, osseointegration, photobiomodulation

## Abstract

**Objective:**

This study evaluated the effects of dual‐wavelength photobiomodulation (PBMT) (infrared and red laser) on osseointegration and bone structure in areas grafted with deproteinized bovine bone (DBB).

**Methods:**

Sixty‐four rats were randomly distributed into four groups. The groups were divided according to the irradiation protocol applied: CTR—Implants not submitted to PBMT; IRL—Implants treated with infrared PBMT; RL—Implants treated with red PBMT; IRL/RL—Implants treated with red and infrared PBMT. The animals underwent grafting procedures on the tibias, and 60 days later, the implants were installed. After the implant placement, PBMT was performed in the PBMT groups through the application of 4 J around the implants. After 15 and 45 days, the animals were euthanized (*n* = 8). Analyses were performed on the amount of mineralized tissues around the implants (BV/TV%), bone tissue microstructure (thickness, space and number of trabeculae—Tb.Th; Tb.Sp, and Tb.N), and osseointegration (bone/implant contact—%BIC and Bone between the threads—%BBT).

**Results:**

The IRL group presented higher values of BV/TV%, while the RL group presented higher values of Tb.N when compared with the CTR group at 45 days (*p* < 0.05). The IRL/RL group presented higher values of BV/TV% and Tb.N when compared with the CTR group at 45 days (*p* < 0.05). Although no statistical differences were found, the IRL/RL group presented higher %BIC and %BBT.

**Conclusion:**

It can be concluded that the combination of red and infrared lasers does not improve the osseointegration of the implants installed in DBB‐grafted areas of rat tibiae.

## 1 Introduction

Bone defects resulting from tooth loss, tumor removal, or trauma impact oral rehabilitation after osseointegrated implants [1]. Although implants with special configurations present great predictability in the treatment of edentulism in areas with a limited amount of native bone [2, 3], guided bone regeneration techniques are still necessary to promote increased bone availability for the correction of these defects and subsequent installation of osseointegrated implants in an ideal tridimensional position [1, 4].

Bone tissue substitutes are essential components for guided bone regeneration techniques. Among these biomaterials, osteoconductive bone substitutes stand out for offering greater patient acceptance and less surgical morbidity, since their use eliminates the need for a second surgical site for autogenous graft harvesting [5, 6]. Deproteinized bovine bone (DBB) is the osteoconductive bone substitute with the greatest available scientific evidence [6, 7]. However, although the use of DBB is associated with favorable clinical results, its biological capacity for bone formation only via osteoconduction is less effective when compared to autogenous bone grafts [8]. Furthermore, in clinical practice, implants installed in areas grafted with osteoconductive biomaterials require a longer period for the establishment of the osseointegration process [9, 10].

Therefore, the combination of osteoconductive bone substitutes with growth factors [11, 12], autogenous bone grafting [5], blood concentrates [13], or low‐power laser photobiomodulation (PBMT) [14] has been proposed to improve bone healing and osseointegration in these grafted areas. PBMT is a therapy that accelerates the repair process through the application of light energy at specific wavelengths and has shown positive effects on bone tissue healing [15]. For low‐intensity applications, two groups of lasers have been used according to their wavelength: red (660 nm) and infrared (830 nm). Lasers with wavelengths within the infrared range have greater penetrability within tissues and are therefore preferable for accelerating bone healing [16, 17]. However, their irradiation protocol requires seven irradiation sessions after the surgical procedure, which may reduce patient adherence to the complete protocol [18]. Single sessions of red laser PBMT have also been shown to be effective in the periodontal regeneration process in preclinical studies in immunosuppressed animals [19, 20]. Furthermore, a study demonstrated that both red laser and infrared laser improved bone repair in postextraction alveolar areas in animals subjected to the use of alendronate [21].

The combination of the two wavelengths for PBMT could be beneficial by promoting healing in both deep and superficial elements of a surgical area [22]. However, direct comparisons of the ability of red and infrared lasers and their combined use to accelerate the osseointegration process of implants in grafted areas still require further investigation. Therefore, the objective of the current study was to evaluate whether the combination of low‐intensity PBMT with infrared and red lasers improves the osseointegration of implants in areas grafted with DBB.

## 2 Material and Methods

This study was submitted to and approved by the Animal Use Ethics Committee of the Federal University of Uberlândia (UFU), School of Dentistry of Uberlândia, Brazil (CEUA/UFU: 011‐20). For this study, 64 rats (Rattus novergicus, Wistar strain) aged 3 months and weighing between 250 and 300 g were used, kept in a controlled environment with a temperature of 21 ± 1°C, humidity of 65%–70%, and light cycles of 12 h. The animals were fed specific rat chow and offered water *ad libitum*. This study was conducted in accordance with the ARRIVE protocol for conducting preclinical studies.

### 2.1 Animals and Groups

Sixty‐four animals were randomly allocated into 4 groups of 16 animals each, divided according to the irradiation protocol applied to the installed implants: CTR—Implants that were not subjected to PBMT; IRL—Implants treated with infrared PBMT; RL—Implants treated with red PBMT; IRL/RL—Implants treated with PBMT combining red and infrared lasers. The animals were subjected to the grafting procedure on the right tibias, and after 60 days, the implants were placed in the grafted area and, simultaneously, the PBMT protocol was carried out. Subsequently, the implants were installed, and the PBMT procedure was initiated in the groups where the irradiation procedure was planned. The animals were euthanized after 15 or 45 days, and samples were collected for microtomographic and histomorphometry analyses.

### 2.2 Grafting Procedure

The animals were anesthetized with a combination of Xylazine (Rompum, Bayer S.A., São Paulo, SP, Brazil) at a dosage of 20 mg/mL/10 mg/kg/IP of body mass, together with Ketamine (Agener União Ltda, São Paulo, SP, Brazil) at a dosage of 50 mg/mL/80 mg/kg/IP 8 mg/kg of body weight. Subsequently, trichotomy of the inner region of the right hind legs was performed, followed by antisepsis. An incision of approximately 10 mm was made in planes over the tibial tuberosity. After delicate dissection, the bone tissue was subjected to osteotomy using a spherical drill mounted at a counter‐angle with the aid of an electric motor set at 1200 rpm (BLM 600—Driller, São Paulo, SP, Brazil) under abundant irrigation with sterile saline solution. The final measurements of the defect were 4 mm in length and width and 1.5 mm in depth, which was then filled with DBB (Cerabone, Botiss, Zossen, Germany). The tissue was sutured immediately over the grafted area internally with 5.0 absorbable thread (Vicryl Ethicon, Johnson & Johnson, São José dos Campos, Brazil) and externally with 4.0 nylon thread (Ethicon, Johnson & Johnson, São José dos Campos, Brazil), after which the area was left for a period of 60 days before the implant surgery. Immediately following the procedure, the animals received intraperitoneal penicillin associated with streptomycin at a dosage of 0.8 mL/kg of weight in a single dose (Multibiótico Small, Vitalfarma, São Sebastião do Paraíso, MG, Brazil) and 1 mg/kg of weight of ketoprofen 1% (Ketoflex; Mundo Animal, São Paulo, Brazil) for 3 days every 24 h.

### 2.3 Implant Installation Procedure

After a period of 60 days, surgery was performed to place the implants. An incision like the first surgical procedure was made in the tibial tuberosity. Confirmed the osseointegration of the biomaterial clinically by the complete filling of the defect by mineralized tissue, the grafted region was prepared for implant installation by applying a progressive sequence of drills (spear drill; 2.0 mm spiral drill—Neodent®; Curitiba, PR, Brazil) to accommodate a titanium implant 4 mm high and 2.2 mm in diameter (machined surfaces, Curitiba, PR, Brazil). All drilling was performed with the aid of an electric motor, set at 1200 rpm, under abundant irrigation with sterile saline solution. The implant was installed using a digital wrench (1.2 mm hexagonal digital wrench—Neodent, Curitiba, PR, Brazil. Tissue suturing and the postoperative medication protocol were similar to those used in the initial surgery. Fifteen or forty‐five days after the implant placement procedure, the animals were euthanized by an overdose of anesthetic.

### 2.4 PBMT Therapy

An InAlA/GaAlA laser (Therapy EC 100 mW, ϕ ∼0.600 μm, tip divergence = 0.37 rad, CW, spot area of 0.0283 cm^2^, DMC Equipamentos, São Carlos, SP, Brazil) was used to perform the irradiations. This device operates in red (*λ* 660 nm), infrared (*λ* 808 nm), or combined (*λ* 660‐λ 808 nm) irradiation modes. The device was positioned perpendicular to the implant axis. After tissue suturing, the tip of the device was placed in direct contact with the animal's skin. The laser was applied at four points around the implant to cover all adjacent tissue. This protocol aimed to enhance soft tissue healing and minimize the risk of bone tissue exposure. The laser was irradiated in a single session trans‐surgically immediately after the implant placement for all the animals in the groups with this therapy. The irradiation energy density used was approximately 35.33 J/cm^2^/point, totaling 141.32 J/cm^2^ per session. The emission of 1 J of energy took 10 s for PBMT with the red or infrared laser and 5 s with the combined application of the two wavelengths. This resulted in 40 s per session on each animal with the red and infrared lasers applied separately and 20 s when both wavelengths were applied in combination.

### 2.5 Microtomographic Analysis

The tibiae were fixed in 4% paraformaldehyde for 48 h and subsequently stored in 70° alcohol. These samples were scanned using a SkyScan 1272 microtomograph (Bruker, Kontich, Belgium) with the following parameters: Camera pixel: 12.45; X‐ray tube power: 65 kVP, X‐ray intensity: 385 μA, integration time: 300 ms, filter: Al‐1 mm, and voxel size: 18 μm^3^. Initially, software (NRecon, Data Viewer) was used for three‐dimensional reconstruction and spatial repositioning of the images, enabling the acquisition of the coronal plane of each sample. Different software (CTAnalyser, Aatselaar, Belgium) was then utilized for the actual analysis of the images. The first step was to export the images obtained by the first software. Next, 100 slices were selected from each image to determine the area that was analyzed by the software. The region of interest (ROI) was defined as a circular region with a 0.5 mm margin around the entire diameter of the implant, thus obtaining a geometric figure of a cylinder along the implant. This ROI was defined as total volume (0.5 mm margin around the implants—4.5 x 3.2 mm). As the implants placed did not receive the cover screw, in some cases, a mineralized structure formed within the prosthetic platform. To prevent this region from interfering with the analysis of the mineralized tissue volume around the implant, a second ROI was defined to remove the platform volume. With the results obtained in the two ROIs, it was possible to define the volume of mineralized tissue using the formula: Total Volume – Platform Volume = Volume of mineralized tissue. After selecting the area of interest, a threshold was applied (60–40 shades of gray) that differentiates the implant from the mineralized tissue, and from this separation, the software can calculate, within the selected area, the volume of the mineralized tissue in relation to the total area (BV/TV%) and additional values relating to the number, thickness, and separation of bone trabeculae (Tb.N, Tb.Th, Tb.Sp) were also obtained.

### 2.6 Histomorphometric Analysis

After µCT scanning, the samples were dehydrated in increasing series of ethanol (70°–100°) and infiltrated and polymerized using light‐curing resin (Technovit 7.200 VLC, Kultzer Heraus GmbH & CO, Wehrheim, Germany). The blocks containing the implant and bone tissue were cut at a central point using a cutting and grinding system for nondemineralized samples (Exakt Apparatebau, Hamburg, Germany). The final sections were approximately 45 μm thick and were stained with Stevenel blue associated with fuchsin acid. The sections were then analyzed using an optical microscope (DIASTAR—Leica Reichert & Jung products, Wetzlar, Germany) at 100 × magnification. Histomorphometric evaluations were performed using image analysis software (ImageJ, San Rafael, CA, USA). Using a tracing tool, a linear measurement corresponding to the implant surface across the first six threads bilaterally was obtained. Subsequently, tracing was performed only in the regions where bone tissue was in direct contact with the implant surface. These two measurements allowed for the calculation of the bone‐to‐implant contact (%BIC), defined as the ratio of the bone‐contact length to the total traced surface. Additionally, the same tool was used to measure the total area of the space between the first six threads bilaterally. The bone tissue present within this space was then traced. With these two area measurements, the bone between threads (%BBT) was calculated as the ratio of the bone tissue area to the total area between the threads. These analyses were performed by a single‐trained, blinded examiner (L.S.G.P.).

### 2.7 Sample Calculation

The %BIC data were considered the primary variable of this study. For the sample size calculation, data were considered from the histometric analysis of %BIC in a previous study that evaluated the effect of infrared PBMT on the osseointegration in grafted area with DBB and biphasic ceramics [14]. A minimum difference of 12.85 with a standard deviation of 4.83 was observed between the groups, where statistically significant differences occurred, thus, a sample size of eight animals per group was determined as sufficient for the application of statistical tests, with a type *α* error of 0.05 and a *β* power greater than 0.90.

### 2.8 Statistical Analysis

The data in this study were numerical, and their normal distribution was confirmed by the Shapiro–Wilk test. The data between groups were compared using the two‐way analysis of variance (ANOVA) test supplemented by the Tukey test, where the effects of time and treatments were associated. GraphPad Prism 9 software (San Diego, USA) was used for inferential analysis of the data from this study. All tests were applied at a 95% confidence level.

## 3 Results

### 3.1 Microtomographic Analysis

**Figure 1:**
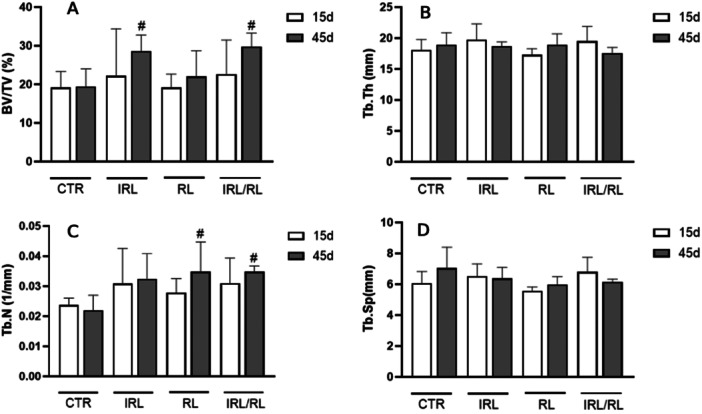
Representative graphs of mean and standard deviation. (A) BV/TV (%); (B) Tb. Th (mm); (C) Tb. N (1/mm); (D) Tb. Sp (mm). ^#^ *p* < 0.05 statistical difference when compared to the CTR group—Two‐way ANOVA supplemented by Tukey's test.

Table 1 shows the mean and standard deviation data for BV/TV%, Tb.Th (mm), Tb.N(1/mm), and Tb.Sp(mm). No statistical differences were found between the groups at 15 days. On day 45, the IRL group (28.60% ± 4.15%) presented higher values of BV/TV%, while the RL group (0.034 ± 0.009 1/mm) presented higher values of Tb.N when compared to the CTR group (19.40 ± 4.61%; 0.022 ± 0.004 1/mm) (*p* < 0.05). On day 45, the IRL/RL group presented higher values of BV/TV% (29.80 ± 3.49%) and Tb.N (0.034 ± 0.001 1/mm) compared to the CTR group (*p* < 0.05). Treatment and time significantly influenced the BV/TV% and Tb.N parameters (*p* < 0.05) (Figure 1).

**Table 1:** Mean and standard deviation data for BV/TV%, Tb.Th (mm), Tb.N (1/mm), and Tb.Sp (mm) in all groups and evaluation periods.

Period	Treatments	BV/TV	Tb.Th	Tb.N	Tb.Sp
15 days	CTR	19.20 ± 4.14	18.08 ± 1.70	0.023 ± 0.002	6.06 ± 0.76
	IRL	22.20 ± 12.19	19.77 ± 2.53	0.030 ± 0.011	6.52 ± 0.80
	RL	18.80 ± 3.96	17.32 ± 0.95	0.027 ± 0.004	5.56 ± 0.26
	IRL/RL	22.60 ± 8.87	19.49 ± 2.39	0.030 ± 0.008	6.80 ± 0.94
45 days	CTR	19.40 ± 4.61	18.90 ± 1.96	0.022 ± 0.004	7.05 ± 1.34
	IRL	28.60 ± 4.15*	18.71 ± 0.67	0.032 ± 0.008	6.38 ± 0.70
	RL	22.00 ± 6.67	18.96 ± 1.74	0.034 ± 0.009*	5.98 ± 0.49
	IRL/RL	29.80 ± 3.49*	17.56 ± 0.95	0.034 ± 0.001*	6.15 ± 0.18

* *p* < 0.05 statistical difference when compared to the CTR group—Two‐way ANOVA supplemented by Tukey's test.

### 3.2 Histomorphometry Analysis

**Figure 2:**
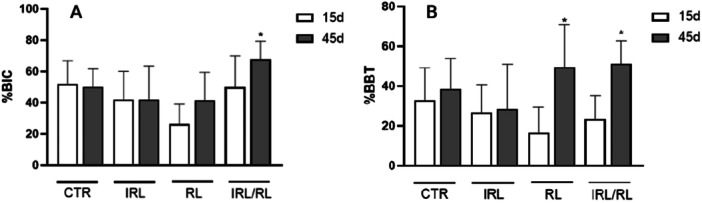
Representative graphs of mean and standard deviation. (A) %BIC; (B) %BBT. *p < 0.05 statistical difference when compared to the same group at 15 days—Two‐way ANOVA supplemented by Tukey's test.

**Figure 3:**
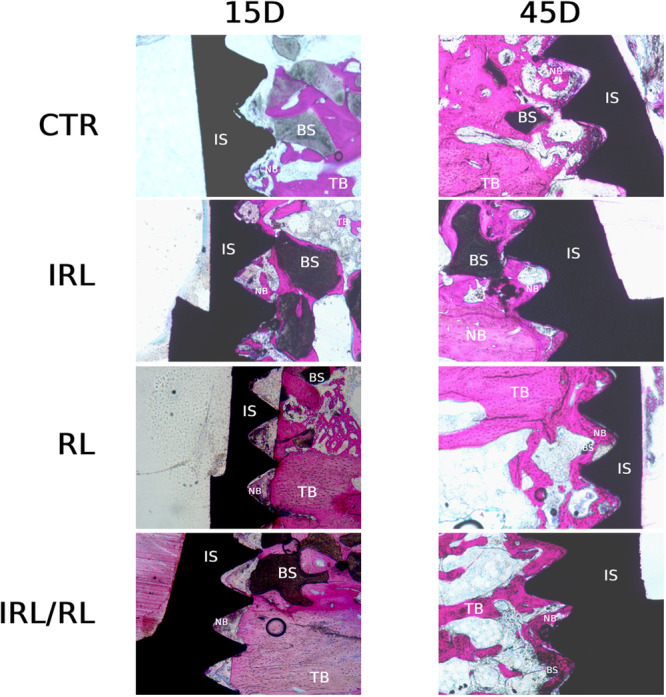
Representative images of the histomorphometric analyses in all groups and evaluation moments. BS, bone substitutes; IS, implant surface; NB, new bone; TB, tibia bone.

Table 2 presents the mean and standard deviation data for %BIC and %BBT. Although higher %BIC and %BBT values were found on day 45 in the IRL/RL group when comparing all groups, no statistical differences were found between treatments (*p* > 0.05). Regarding the evaluation period, the IRL/RL 45 days group presented higher %BIC and %BBT values compared to the IRL/RL 15 days group (*p* < 0.05). Furthermore, the RL 45 days group showed higher %BBT values when compared to the RL 15 days group (*p* < 0.05) (Figures 2 and 3).

**Table 2:** Mean and standard deviation data for %BIC and %BBT in all groups and evaluation periods.

Parameter	Treatment	Period	
		15 days	45 days
%BIC	CTR	51.83 ± 14.97	50.40 ± 11.28
	IRL	42.00 ± 18.04	42.00 ± 21.34
	RL	26.40 ± 12.72	41.50 ± 17.86
	IRL/RL	48.67 ± 18.04	67.14 ± 12.60*
%BBT	CTR	32.83 ± 16.31	38.50 ± 15.40
	IRL	26.60 ± 14.19	28.50 ± 22.37
	RL	16.60 ± 12.86	49.50 ± 21.25*
	IRL/RL	23.38 ± 11.75	51.14 ± 11.67*

* *p* < 0.05 statistical difference when compared to the same group at 15 days—Two‐way ANOVA supplemented by Tukey's test.

## 4 Discussion

The objective of the current study was to evaluate whether the combination of low‐intensity PBMT with infrared and red lasers improves the osseointegration of implants in areas grafted with DBB in a single session. The findings of this study showed that PBMT with simultaneously emitted red and infrared light in a single intraoperative session did not improve the osseointegration of implants placed in areas grafted with DBB. Nevertheless, the investigated protocol demonstrated a higher percentage of mineralized tissue around the implant when compared to the control group.

The microtomographic analysis showed that the IRL group presented higher values of BV/TV% when compared to the CTR group, while the RL group presented higher values of Tb.N when compared to the CTR group. PBMT with red and infrared wavelengths has previously been shown to improve bone healing in grafted and nongrafted bone defects [15, 18, 23, 24]. These effects are associated with PBMT stimulation of mitochondrial chromophores, which accelerate cellular metabolism by stimulating cellular respiration [1]. In mesenchymal cells, which are highly involved in the repair process [25, 26, 27], these effects are more exuberant, since acceleration of the differentiation process can strongly impact the tissue repair process [26, 28]. In relation to bone tissue, PBMT with both wavelengths stimulated osteoblast formation and function, inducing greater production of bone tissue matrix [25, 28]. These effects may justify the microstructural alterations observed in the current study after PBMT with red and infrared lasers applied separately in areas grafted with DBB.

An important finding in the current study is that dual‐wavelength PBMT increased the number of trabeculae and the amount of mineralized tissue without, however, interfering with the space and thickness of the trabeculae. It is likely that these variables are more affected by the structure of the bone substitute used, which, due to its low resorption rate, maintains its trabecular thickness even as the healing process progresses [18]. Furthermore, the fact that there is a greater amount of mineralized tissues does not necessarily mean that the newly formed bone is of good quality. Extremely mineralized bone with high trabecular density represents bone with lower vascularization and low compressive strength [29, 30]. On the other hand, bones with low mineral content and less trabeculae are more prone to fractures [31]. Further studies are needed to verify whether adjunct therapies applied to improve the repair process of the grafted area would result in better osseointegration of the implants.

Although not statically significant, the combined use of PBMT with the two wavelengths increased %BIC and %BBT values when compared to the other groups. Regarding the evaluation period, it was found that the IRL/RL 45 days group presented better %BIC and %BBT values when compared to the IRL/RL 15 days group (*p* < 0.05). The reason for combining lasers with different wavelengths is that the red laser is better absorbed superficially, while the infrared laser has greater tissue penetration [16, 17, 23], improving healing at the different parts of the wound [32]. Indeed, previous clinical studies demonstrated that the combined use of red and infrared lasers in PBMT therapy improved bone tissue formation [23] and soft tissue formation [22] associated with third molar removal [33]. On the other hand, another clinical study showed that the use of dual‐wavelength PBMT did not influence the stability of dental implants but improved soft tissue healing up to 8 weeks after the implant placement procedure [32]. It is possible that the differences in the PBMT protocol can explain the different findings between the studies.

Another intriguing outcome was that the %BIC and %BBT values in the RL group were lower than the other groups at 15 days, despite not presenting a statistical difference. A possible reason for this finding is the low tissue penetrability of the red laser and, consequently, higher accumulation of energy in the superficial region of the peri‐implant [16, 17]. Thus, some reasons for these findings can be hypothesized, may not have irradiated with enough energy in deeper regions of the grafted area around the implant [16], and a single session of laser therapy was not able to provide enough energy to influence the osseointegration process [14, 23].

A previous study demonstrated that a high energy density (> 20 J/cm^2^) led to significantly reduced cell proliferation, migration, and metabolism [34]. It is important to highlight that previous studies in the literature demonstrate that energy delivered over an extended period—corresponding to a lower energy density—exerts a biostimulatory effect on bone tissue, enhancing osteoblastic proliferation and cell differentiation. In this study, the simultaneous application of red and infrared lasers resulted in a higher energy density compared to their isolated use. This occurred because the emitted energy remained constant, whereas the irradiation time for the combined protocol was reduced by half [35, 36]. However, the energy density in our study has not change the osseointegration in grafted area since the there were no differences between the PBMT with red and infrared laser used combined or isolated.

A limitation of this study was the inability to perform a histological evaluation of the grafted site, as this procedure could compromise the implant's primary stability. However, a previous preclinical study of our group evaluates the effect of the similar PBMT protocols used in this study in the bone repair in DBB‐grafted areas in rats mandible. The PBMT therapy was applied after the grafting procedures. This study showed that the IRL/RL PBMT improved the bone formation, but the DBB particles remnants was observed in all the groups [22]. Indeed, the remaining DBB particles were clinically observable during implant placement. The presence and density of these particles may affect the laser's penetration depth; therefore, further studies are necessary to confirm this hypothesis. An important fact is that this study presented a reduced number of PBMT sessions (1 session immediately after the implant placement), and the dosage of energy emitted to the recipient bed can impact the results of PBMT [35, 36]. In fact, preclinical studies which demonstrated that Red or Infrared PBMT increases bone formation and osseointegration in grafted areas included seven PBMT sessions [14, 18, 21]. Further studies are necessary to define the dosage, number of sessions, and optimal timing for PBMT application to optimize bone formation in both grafted and nongrafted bone defects. Further studies are needed to define the dosage, the number of sessions, and the ideal timing for the application of PBMT and to verify through microtomography and histomorphometry the conditions of bone repair in the grafted area that will receive the implant.

## 5 Conclusion

Within the limitations of this preclinical study, it can be concluded that the PBMT with combination of red and infrared laser irradiated in a single session does not improve the osseointegration of the implants installed in DBB‐grafted areas of rat tibiae.
